# Incidence and Risk Factors of 30-Day Surgical Site Infection after Primary Total Joint Arthroplasty in a Middle-Income Country: A Single-Center Experience

**DOI:** 10.3390/ijerph18030863

**Published:** 2021-01-20

**Authors:** Vuk Marusic, Ljiljana Markovic-Denic, Olivera Djuric, Andja Cirkovic, Vladimir Nikolic, Emilija Dubljanin-Raspopovic, Marko Kadija

**Affiliations:** 1 Institute of Epidemiology, Faculty of Medicine, University of Belgrade, 11000 Belgrade, Serbia; lj.denic@gmail.com (L.M.-D.); nikolicvladimir32@gmail.com (V.N.); 2 Section of Public Health, Center for Environmental, Department of Biomedical, Metabolic and Neural Sciences, Nutritional and Genetic Epidemiology (CREAGEN), University of Modena and Reggio Emilia, 41121 Modena, Italy; oliveradjuric87@gmail.com; 3 Epidemiology Unit, Azienda Unità Sanitaria Locale—IRCCS di Reggio Emilia, 42122 Reggio Emilia, Italy; 4 Institute of Statistics and Informatics, Faculty of Medicine, University of Belgrade, 11000 Belgrade, Serbia; andja.aleksic@gmail.com; 5 Faculty of Medicine, University of Belgrade, 11000 Belgrade, Serbia; edubljaninraspopovic@gmail.com (E.D.-R.); kadija.marko@gmail.com (M.K.); 6 Clinic for Physical Medicine and Rehabilitation, Clinical Center Serbia, 11000 Belgrade, Serbia; 7 Institute for Orthopedic Surgery and Traumatology, Clinical Centre of Serbia, 11000 Belgrade, Serbia

**Keywords:** surgical site infection, total hip arthroplasty, total knee arthroplasty, prospective cohort study, risk factors

## Abstract

The data about the incidence and risk factors for surgical site infections (SSIs) following total joint arthroplasty (TJA) in middle-income countries are still scant. The aim of this study was to assess the incidence and risk factors associated with 30-day SSIs following total hip arthroplasty (THA) and total knee arthroplasty (TKA). The study was conducted at the Clinic for Orthopedic Surgery and Traumatology, Clinical Center of Serbia (CCS) in Belgrade, from May 2016 to April 2018. All patients undergoing THA or TKA were followed throughout hospitalization until day 30 after discharge. Of the 1073 admitted patients, 459 had THA and 230 had TKA. The incidence rate of surgical site infections (SSIs) among the patients who underwent THA was 5.4%, which is 3.8 per 1000 postoperative patient-days, while the rate among those who had TKA was 4.8%, i.e., 3.4 per 1000 postoperative patient-days. Out of the 36 SSIs, 15 were deep and 21 were superficial incisional ones. Among the variables examined, the independent risk factors for SSIs after THA were the American Society of Anesthesiologists (ASA) score > 2 (RR = 3.17; 95% CI—1.26–8.02), smoking (RR = 3.14; 95% CI—1.26–7.82) and peripheral vascular disease (PVD) (RR = 6.09; 95% CI—2.35–15.77), and after TKA, only PVD (RR = 3.87; 95% CI—1.09–13.76) was the risk factor. Incidence rates of SSIs after arthroplasty are higher compared to reports from developed countries. Therefore, it is necessary to enhance infection prevention and control measures with strict control of modifiable risk factors.

## 1. Introduction

Surgical site infections (SSIs) still represent important adverse postoperative events in orthopedic surgery [1]. Although SSIs following total joint arthroplasty (TJA) are not so frequent, they are associated with severe complications that can subsequently require a longer postoperative hospital stay, rehospitalization, reoperation, temporary prosthesis removal, and prolonged antimicrobial therapy. Furthermore, they can reduce patient functionality, increase mortality, and lead to excess treatment costs [2].

A significant difference in the incidence of TJA SSIs was noted between the developed, low-income, and middle-income countries [3]. After decades of rising incidence, SSIs showed a decreasing trend in the developed countries due to the bundle of applied preventive measures and advances in surgical techniques [4]. However, they still represent an important problem in the developing countries, which is a consequence of limited resources, suboptimal practices, and lack of adherence to the infection control guidelines [5].

More than a decade ago, the European Center for Disease Prevention and Control (ECDC) prepared the protocol for SSI surveillance as an effort to unify data collection and reporting procedures of SSIs in the European Union countries and to define the risk factors of SSIs for the most important types of surgical procedures. This Center has been annually publishing the data on SSIs in the EU countries [6]. However, to the best of our knowledge, there are very few publications from non-EU Southern European countries that assessed the incidence and risk factors of SSI in orthopedic surgery [7,8,9], especially for SSIs after TJA [10]. 

Therefore, the aims of this study were (a) to estimate the incidence rate of SSIs after total hip and total knee arthroplasty for 30 days after the operation and (b) to determine the risk factors associated with these infections.

## 2. Materials and Methods

### 2.1. Study Design and Patient Selection

A prospective cohort study was conducted at the Clinic for Orthopedic Surgery and Traumatology, Clinical Center of Serbia (CCS) in Belgrade, from May 2016 to April 2018. The eligible cohort of hospitalized patients included all persons older than 18 years undergoing either primary total hip arthroplasty (THA) or primary total knee arthroplasty (TKA). Indications for the implantation of the prosthesis were hip or knee joint degenerative changes. We excluded all patients with immunodeficiency, splenectomy, patients scheduled for radiotherapy or treatment of oncological diseases, as well as patients with whom communication was not possible and those who refused to participate in the study. According to the National Academy of Sciences’ National Research Council’s operative site classification, all wounds were classified as category I, primarily clean wounds [11]. All patients were followed daily throughout the hospital stay by an orthopedic surgeon, an infection control epidemiologist, a doctoral student, and infection control nurse. Patients who were discharged earlier than day 30 after surgery were called for a checkup at a surgical outpatient clinic.

### 2.2. Data Collection and Sources of Data

The data were recorded using a preprinted data collection form which was designed according to the latest available literature. Two primary researchers (L.M.D. and V.M.) searched studies reporting risk factors of SSIs in the sphere of orthopedy. The data collection form was conceived by the study multidisciplinary team using literature data about cohort studies, a systematic review of risk factors, and meta-analysis [12,13]. Besides that, available data from the patients’ medical records, operative and anesthesia lists, and patient medication charts at our hospital were taken into account. To ensure accuracy of the data, a pre-test of the data collection form was done on a certain number of respondents that were not included in the analysis. Afterwards, further modification of the data collection form was carried out by the study team.

All patients were followed daily throughout the hospital stay by an orthopedic surgeon, an infection control epidemiologist, an infection control nurse, and a doctoral student who prospectively collected the data using the above-mentioned data collection form. Patients were monitored until discharge and on the 30th day after the operation within the regular control examination. Before the discharge, they would receive the exact time of the examination. Those who did not come for the examination on the scheduled day were called by phone as a reminder. For the patients who did not respond, multiple contacts were attempted. In this way, the information on the eventual death of a patient was obtained from the patient’s proxy.

The data collection form included the data on demographic characteristics of patients (sex, age, type of residential environment—rural vs. urban), comorbidities (heart disease, acute myocardial infarction, hypertension, peripheral vascular disease, cerebrovascular insult, diabetes mellitus, anemia, rheumatoid disease, chronic kidney disease, urinary disorder), Charlson comorbidity index (CCI), smoking and alcohol habits, and body mass index (BMI) estimated in kg/m^2^. The CCI is comprised of 17 different items divided into four groups which are weighed/scored differently with one, two, three, or six points so that the final score ranges from 0 to 30 points [14]. Preoperative data included preoperative bathing, shaving of the patient’s operational field, and surgical antimicrobial prophylaxis (SAP). The SAP was considered adequate if it was administered within 1 h before the incision and was not prolonged more than 24 h after the operation. The patients’ ASA scores of the physical status classification system defined by the American Society of Anesthesiologists as an index for the severity of the underlying disease [15] were obtained from anesthesiologic lists. This score has six degrees (I-VI) which are defined as follows: ASA I—a normal or healthy patient, ASA II—a patient with mild systemic disease, ASA III—a patient with severe systemic disease, ASA IV—a patient with severe systemic disease that is a constant threat to life, ASA V—a moribund patient who is not expected to survive without operation, and ASA VI—a declared brain-dead patient whose organs are being removed for donor purposes. But the 6th degree is not used for assessing patients in clinical settings, because patients are still alive. We divided patients into two groups according to the obtained ASA scores into those with ASA ≤ 2 and those whose ASA score was higher than 2.

Other data about the surgical procedure, the type of applied anesthesia, the duration of the operative procedure, the number of persons in the operating room, the transfusion, and the assessment of the general condition of the patient were obtained from operative and anesthesiologic documents. 

For each patient, the National Healthcare Safety Network (NHSN) SSI risk index was calculated. It ranges from 0 (lowest risk) to 3 (highest risk), and it consisted of three different variables (ASA score, surgical wound classification/contamination, and procedure duration in minutes). Each variable could be scored 0 or 1. Variables were scored with 1 point if the ASA score was 3 or higher if the wound was contaminated or dirty (class III or IV) and if the operative procedure lasted longer than the cut-off point (75th percentile) [16].

The presence of SSI was prospectively assessed by daily wound assessment, clinical course, and microbiological results by infection control epidemiologists and attending surgeons.

### 2.3. SSI Definition

All SSIs were diagnosed based on the recommendations of the Centers for Disease Control and Prevention (CDC) in Atlanta, USA, translated into the Serbian language. The primary classification of SSIs was performed based on anatomical localization, according to which they were divided into three categories: superficial incisional, deep incisional, or organ/space infections.

### 2.4. Outcome

The outcome variable of the study was the occurrence of SSI after THA and SSI after TKA developed within 30 days after the implantation of prosthetic joints of the hip or knee. 

### 2.5. Statistical Analysis

In the statistical analysis of collected data, descriptive and analytic statistics were used. In descriptive statistics, discontinuous variables were represented by numbers and percentages, while continuous variables were represented by means and standard deviations (SD) or by the median interquartile range (IQR). All continuous variables were previously tested using the Kolmogorov–Smirnov test to determine normality of data distribution. Patients were categorized as those with or without SSI after TKA and after THA. Analytical statistics implied the use of the Student’s t-test and of the Mann–Whitney U test in the analysis of continuous variables, while the Pearson’s chi-squared test and the Fisher’s exact test were used to compare the data among the examined patient groups. To calculate relative risks (RRs) as individual SSI predictors during hospitalization and assess their impact on SSI development, as well as 95% CI, univariate analysis and multivariate logistic regression were performed. All variables with the significance level *p* < 0.1 in the univariate analysis were involved in the multivariate analysis model, and several variables whose significance was *p* > 0.100 and which are clinically important and could be predictors of the development of SSIs were included in the final model. The ASA score, Charlson comorbidity index, body mass index, peripheral vascular disease smoking, NHSN risk index, duration of surgery, number of blood units given to a patient during surgery were used as predictors of SSIs. All variables were regressed on SSI with adjustment for patient characteristics, age, and sex. We made two models of regression analysis, one for THA and the other for TKA. Both models were adjusted for age and sex as confounders in the multivariate analysis and eliminated using Wald backward method. All tests were two-sided, and all variables with a *p*-value < 0.05 were considered statistically significant.

Multicollinearity diagnostics was assessed using the variance inflation factor (VIF) with cut-off > 5 [17]. Since there was no collinearity, no effect modifiers were detected.

The entire statistical analysis was carried out using the statistical software package SPSS for Windows version 17.0 (IBM-SPSS Inc., Armonk, NY, USA).

### 2.6. Ethical Principles

The study was approved by the Ethical Committee of the Medical School, University of Belgrade, No. 29/III-20 from 28 March 2016, and by the Ethical Board of the Clinical Center of Serbia, No. 124/9 from 23 June 2016, and subsequently conducted according to all ethical principles. Each aim and method of the study was explained in detail to patients or their legal guardians before any data collection and study onset.

## 3. Results

### 3.1. Sociodemographic Characteristics of THA and TKA Patients

**Figure 1:**
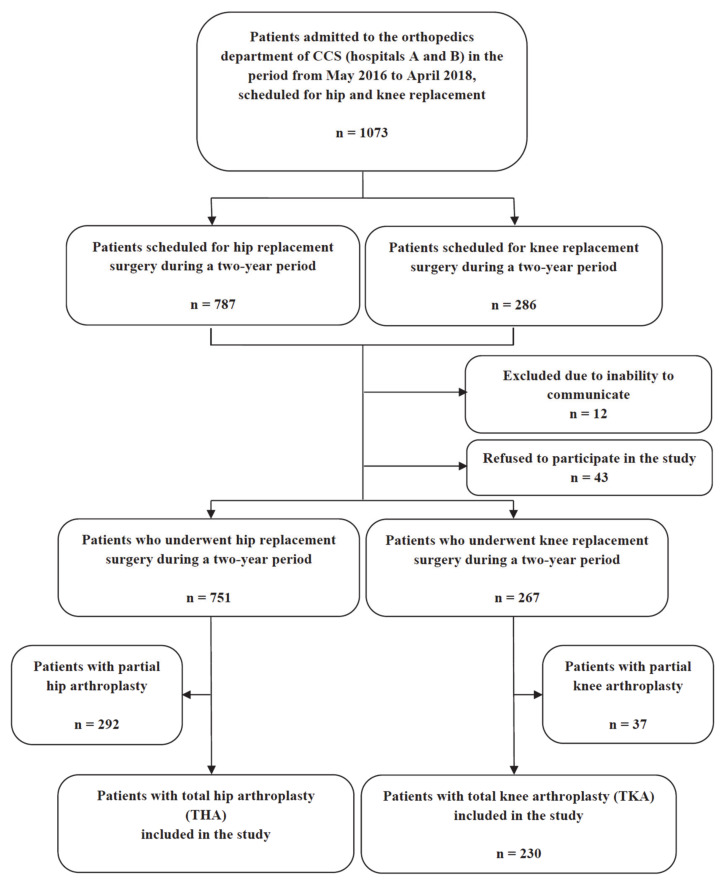
Flowchart of the study.

During the study period, 1073 patients were admitted to the Clinic for Orthopedic Surgery and Traumatology, CCS, of which 787 were scheduled for primary hip replacement surgery and 286—for knee replacement surgery. Twelve patients were excluded due to their inability to communicate, and 43 patients refused to participate in the study. Further, 292 patients with partial hip arthroplasty and 37 patients with partial knee arthroplasty were excluded. Thus, 1018 patients were included in the study: 459 patients who underwent primary THA and 230 patients who underwent primary TKA. The flowchart of patient selection is depicted in Figure 1.

The baseline patients’ characteristics are presented in Table 1. The male/female ratio was slightly higher among THA patients (0.74 vs. 0.52) and a higher proportion of these patients lived in urban areas (69.9% vs. 57.4%, *p* < 0.001). The average age of all patients was 69.2 ± 10.9 years; however, TKA patients were older (67.82 ± 7.93 vs. 64.88 ± 10.59; *p* < 0.001). These patients had two or more comorbidities more often than THA patients.

The most prevalent comorbidity in both groups was hypertension (69.3% of THA cohort patients and 84.3% of TKA cohort patients, respectively). The second most frequent ones were cardiovascular events (AMI, heart disease, or rhythm disorders): 47.9% of THA patients and 53% of TKA patients (not presented in Table 1). Overall comorbidity was also assessed using the Charlson comorbidity index, which was higher in TKA patients (*p* = 0.036). The mortality rate in both groups was the same.

SSI occurred in 36 patients (5.22%). The rates of total SSI were 5.4% or 3.8 per 1000 post-operative patient-days for THA and 4.8% or 3.4 per 1000 post-operative patient-days for TKA. There was no difference in rates between patients who underwent THA and patients who underwent TKA (*p* = 0.712).

Out of the 36 SSIs, 15 were deep incisional infections: 9 occurred after THA, with the incidence of 1.9%, and 6—after TKA, with the incidence of 2.6%. The other 21 SSIs were superficial incisional infections: 16 occurred after THA and 5—after TKA, with the incidence of 3.4% and 2.1%, respectively.

### 3.2. Risk Factors of Surgical Site Infections among THA and TKA Patients

Results of univariate and multivariate logistic regressions for THA and TKA are presented in Table 2. The potential predictors of SSIs after THA were the ASA score over 2, longer surgical procedures, smoking habit, presence of peripheral vascular disease, greater BMI, NHSN risk index 2, greater Charlson comorbidity index, and a greater number of blood units given during the surgical intervention. Multivariate regression left the ASA score over 2, smoking habit, and presence of peripheral vascular disease as independent risk factors for SSIs after THA. Patients with the ASA > 2 as well as smokers had a three times greater risk of SSIs after total hip arthroplasty. Peripheral vascular disease represented a factor with the greatest impact on the risk of SSIs after total hip arthroplasty. Patients with peripheral vascular disease had a six times greater risk than those without this condition. 

On the other hand, the presence of peripheral vascular disease was the only independent risk factor of SSIs after TKA. Patients with peripheral vascular disease had an almost four times higher risk of suffering SSIs after total knee arthroplasty regardless of age and gender.

However, the variables not significantly associated with SSIs were as follows: patient characteristics (age and gender), coded comorbidities, biochemical parameters (urea, creatinine, hemoglobin, CRP), preoperative factors (remote healthcare-associated infections (HAI) before the operation, length of stay before the operation—in days, preoperative bathing performed, preoperative shaving performed, time of preoperative shaving before the procedure—in hours, surgical antibiotic prophylaxis (divided into three categories: a single dose given 60 min prior to the operation, prophylaxis on the day of surgery and for > 1 post-operative day)), operative factors (number of persons in the operating room, type of anesthesia), postoperative factors (time of the first wound toilette, length of wound drainage) for both types of surgery. Besides, the ASA score (≤ 2 vs. > 2), the Charlson comorbidity index, the BMI, the number of blood units given during the operation, and the NHSN risk index were non-significantly associated with SSIs after TKA (not presented in the table).

### 3.3. Microorganisms Isolated from Surgical Wounds

A total of 45 microorganisms were isolated from 36 SSIs. Out of those registered after TJAs, the most frequent microorganism was coagulase-negative staphylococci (CoNS) (26.7%), of which 58.3% were methicillin-resistant, followed by *Acinetobacter* spp. (24.4%) and *Staphylococcus aureus* (22.3%), of which 70% were methicillin-resistant (MRSA). All *Acinetobacter* isolates were resistant to carbapenems (Table 3).

## 4. Discussion

This study showed that the incidence rate of SSIs among patients who underwent THA was 5.4%, which is 3.8 per 1000 postoperative patient-days, while the rate among those who had TKA was 4.8%, i.e., 3.4 per 1000 postoperative patient-days. These rates are higher than the incidence rate obtained from the surveillance system in the EU countries. Namely, the average incidence rate of SSIs after all hip prosthesis surgeries in the EU was 1.0% with the variation among countries from 0.4% to 2.2%, and the incidence density rates were 0.3 (0.2–0.9) per 1000 postoperative patient-days. The SSI rates after knee surgery were 0–5% (0.2–2.7%) and 0.1 (0.1–0.5) per 1000 postoperative patient-days [6]. The incidence rates in the US were less than 1% after THA and 1–2% after TKA [11] with a decreasing trend for a decade [4]. In our study, the incidence rate of deep SSIs in patients who underwent TKA was higher than in patients who underwent THA, as found in other studies outside Europe [4]. On the contrary, the frequency of deep SSIs was higher after THA in the EU countries [6]. According to the CDC definitions that were valid when our study started, each patient was followed until the end of the one-year period after the implant had been inserted. In this article, we presented only the incidence of SSIs occurring within 30 days after surgery, although post-discharge surveillance was organized. According to our non-published data, it was revealed that only 1.01% more infections were detected within one year after surgery, which is in agreement with the results of other studies [18].

Higher SSI rates after THA and TKA surgery reported in our survey can be explained by the lack of resources, the lack of adequate ventilation in operating rooms, and the lack or incomplete application of infection prevention and control (IPC) measures.

In this study, the independent risk factors of SSI after THA were physical status presented as the ASA score ≥ 2, smoking, and peripheral vascular disease, and after TKA, the only independent risk factor was peripheral vascular disease. The ASA score is often used as an indicator of a patient’s basic health condition before surgery. Although one of the objections may be that it is a subjective assessment, it should be emphasized that every anesthesiologist is trained to determine this measure. Most studies confirmed that a higher ASA score is associated with the development of SSIs [7,19], although in some studies, this association was not observed [20,21,22].

Tobacco consumption is associated with microvascular constriction, reduced tissue oxygenation, and an increased risk of postoperative wound complications [23]. According to the results of the meta-analysis of periprosthetic joint infections after TJA, smokers had a 1.8 times higher risk of infections than non-smokers [13]. In our study, a much higher percentage of current and ex-smokers was observed among those who developed SSIs after THA. However, it is important to note that some studies have shown that the risk of developing prosthetic joint infections (PJIs) in smokers and non-smokers is the same [24].

Peripheral vascular disease (PDV) was one of the independent risk factors both for THA and TKA surgery in our study. Patients with THA and patients with TKA were 6.4 and 3.5 times more likely to develop SSI, respectively, if they had a history of peripheral vascular disease. It is well-known that reduced tissue oxygenation and extended time for wound healing can be associated with infections. This fact is proven in ankle joint surgery [25], because this joint is thin and less vascularized. The results for THA and TKA surgery are still controversial. Our results are in agreement with studies that showed that PVD is a risk factor for the onset of SSIs [26,27]. They found that delayed wound healing, skin necrosis, and deep infection can be arterial complications after THA and TKA surgery.

The distribution of microorganisms (MO) that cause SSIs varies greatly between many studies, regions, and countries around the world. According to the results of the surveillance system of HAIs in the USA, in 2015–2017, the most commonly registered pathogens isolated from wounds after orthopedic surgical procedures were *S. aureus*, as much as 60.1% of all the pathogens. More than one-third of *S. aureus* isolates were MRSA [28]. In the EU countries, this microorganism was the most frequent causative agent of SSIs after THA and TKA, too [6]. The second most frequent microorganism was coagulase-negative *Staphylococcus* (CoNS) in both studies [6,28]. In our study, CoNS was the most frequent agent, which is in agreement with the study conducted in Poland [29]. *S. aureus* represented the third most common pathogen. Although it is well-known that preoperative *S. aureus* screening is an effective tool in reducing the risk of SSIs [30], this practice has neither been introduced as mandatory throughout the country nor applied in our hospital. The results of the study conducted at the same hospitals one decade ago [9] showed that the most common isolated pathogens were *S. aureus* (28.9%) and *Acinetobacter* spp. (24.1%), while CoNS was found in only 4.8% of the cases after the same operations. An increase in the frequency of CoNS as a causative agent of SSI, especially an increase in its resistance, has been observed in other countries as well [4]. Coagulase-negative staphylococci, with *Staphylococcus epidermidis* as the most prevalent among all CoNS, are part of the normal microbial flora of human skin. Although these microorganisms are often considered contaminants, deep implant infections caused by those pathogens can lead to severe complications, sometimes even to implant removal [31]. In our study, two deep hip and knee surgical site infections were caused by resistant CoNS. Contrary to the developed counties where few reports have addressed *Acinetobacter* in orthopedic surgery [32], in less developed countries, this pathogen is more frequent [33,34]. The SSIs caused by *Acinetobacter* are difficult to treat, especially if it causes deep infections and is multidrug-resistant (MDR). Sixty-three percent of all *Acinetobacter* isolates caused deep SSIs, and all of them were MDR. Active surveillance of SSIs with timely infection control measures could help reduce this problem in our hospitals, as shown in other countries [32].

### Limitations and Strengths of the Study

The basic limitation of this study is associated with data collection in one center, which is the main university center in Belgrade. Thus, that can be subject to selection bias due to the inclusion of the patients with a severe stage of osteoarthritis. Another limitation is that patients were followed only until day 30, not until the 90th day after surgery if the implant was in place, as defined in the CDC recommendations. Because of that, a small amount of SSIs after TJA may have been lost in between. But the strength of the study is that the same infection control doctor (V.M.) made a prospective cohort design for this study and collected all the data. The results of this study may be affected by a set of biases. It is well-known that “lost to follow-up” is the most important bias in prospective cohort studies [35]. To avoid that, every patient was scheduled for a follow-up examination before discharge. Besides, written instructions on the symptoms related to SSI were provided to each patient, as well as the telephone number of the researcher (V.M.) who each patient could address in case of any problem with a wound. If a patient did not come for a regular examination, he/she would be called several times until the patient or one of his/her relatives would answer the call. Therefore, this type of bias was minimized. Another type of bias can be selection bias known as the “non-responder” bias. However, it is very rare in prospective cohort studies such as ours. Data collection and information bias were minimized due to collecting data from medical charts by one trained infection control doctor and rechecked by the researchers. 

## 5. Conclusions

The independent risk factors of SSI after THA were higher ASA score representing a worse physical status of the patient, current smoking or history of smoking, and peripheral vascular disease, but only peripheral vascular disease was a risk factor after TKA. Out of all the MOs isolated from infected wounds after arthroplasties, the most frequent one was CoNS with its increasing antibiotic resistance, especially when it causes deep SSIs.

We found that the incidence rates of SSIs were higher than in the EU countries and in the US. However, considering that higher rates are possibly caused by the lack of resources, adequate ventilation, and appropriate and complete implementation of IPC measures, their future trend could be decreasing after some minor, but determined improvements. Therefore, it is necessary to enhance infection prevention and control measures with strict control of modifiable risk factors.

## Figures and Tables

**Table 1:** Baseline patient characteristics with THA and TKA.

	Patients with THA	Patients with TKA	
	n (%); Mean ± SD		*p*-Value
	n = 459	n = 230	
Gender			
Male	195 (42.5)	79 (34.3)	0.040
Female	264 (57.5)	151 (65.7)	
Age	64.88 ± 10.59	67.82 ± 7.93	<0.001
Type of residential environment			
Rural	138 (30.1)	98 (42.6)	0.001
Urban	321 (69.9)	132 (57.4)	
Coded comorbidities			
0	85 (18.5)	17 (7.3)	0.001
1	139 (30.3)	80 (34.8)	
≥2	235 (51.2)	133 (57.9)	
ASA score			
≤2	310 (67.5)	153 (66.5)	0.789
>2	149 (32.5)	77 (33.5)	
Charlson comorbidity index	0.71 ± 1.05	0.83 ± 1.04	0.036
Mortality rate *	0.44	0.44	

* Per 1000 patient-days of hospitalization.

**Table 2:** Risk factors of surgical site infections (SSIs) after total hip arthroplasty and total knee arthroplasty according to the univariate and multivariate regression analysis.

	Univariate Analysis (*p* < 0.1)			Multivariate Analysis *		
Risk Factor	RR	95% CI	*p*-Value	RR	95% CI	*p*-Value
	SSIs after total hip arthroplasty					
ASA score ≤ 2 vs. > 2	4.86	2.05–11.54	<0.001	3.17	1.26–8.02	0.015
Surgical procedure duration—in minutes	1.01	0.99–1.02	0.096			
Smokers and ex-smokers	3.27	1.45–7.38	0.004	3.14	1.26–7.82	0.014
Peripheral vascular disease	7.33	3.13–17.18	<0.001	6.09	2.35–15.77	<0.001
Body mass index (BMI)	1.08	1.01–1.16	0.033			
NHSN risk index (≤ 1 vs. 2)	3.94	1.24–12.57	0.020			
Charlson comorbidity index	1.86	1.39–2.49	<0.001			
Number of blood units given to a patient	1.20	1.01–1.44	0.039			
	SSIs after total knee arthroplasty					
Peripheral vascular disease	3.41	0.99–11.70	0.051	3.87	1.09–13.76	0.037
Preoperative bathing performed	3.54	0.87–14.35	0.077			

ASA score—American Society of Anesthesiologists physical status classification system; NHSN index—National Healthcare Safety Network SSI risk index. * Method—backward stepwise (Wald). * Adjusted for age and gender.

**Table 3:** Microorganisms causing SSIs.

	All SSIs	SSis after THA	SSIs after TKA
No of SSIs	36 (100)	25 (69.5)	11 (30.5)
	n (%)		
CoN Staphylococcus*	12 (26.7)	8 (24.5)	4 (33.4)
*Acinetobacter* spp.	11 (24.4)	10 (30.3)	1 (8.3)
*Staphylococcus aureus*	10 (22.3)	5 (15.1)	5 (41.7)
*Escherichia coli*	3 (6.7)	3 (9.0)	
*Pseudomonas aeruginosa*	3 (6.7)	3 (9.0)	
*Klebsiella*–*Enterobacter* spp.	2 (4.4)	2 (6.1)	
*Enterococcus* spp.	2 (4.4)	1 (3.0)	1 (8.3)
*Streptococcus anginosus*	1 (2.2)	1 (3.0)	
*Stenotrophomonas maltophilia*	1 (2.2)		1 (8.3)
Total	45 (100.0)	33 (100.0)	12 (100.0)

* CoN *Staphylococcu*s—coagulase-negative *Staphylococcus*.

## Data Availability

Data available on request due to restrictions eg privacy or ethical. The data presented in this study are available on request from the corresponding author. The data are not publicly available due to they are part of the larger database which corresponding author still use for statistical analysis and writing of the PhD thesis.
